# Pupil Localisation and Eye Centre Estimation Using Machine Learning and Computer Vision

**DOI:** 10.3390/s20133785

**Published:** 2020-07-06

**Authors:** Wasiq Khan, Abir Hussain, Kaya Kuru, Haya Al-askar

**Affiliations:** 1 Computer Science Department, Liverpool John Moores University, Liverpool L33AF, UK; a.hussain@ljmu.ac.uk; 2 School of Engineering, University of Central Lancashire, Preston PR12HE, UK; kkuru@uclan.ac.uk; 3 Computer Science Department, College of Engineering and Computer Sciences, Prince Sattam Bin Abdulaziz University, Al-Kharj 11942, Saudi Arabia; h.alaskar@psau.edu.sa

**Keywords:** pupil detection, deep eye, iris detection, eye centre localisation, eye gaze, facial analysis, image convolution, machine intelligence, pupil segmentation

## Abstract

Various methods have been used to estimate the pupil location within an image or a real-time video frame in many fields. However, these methods lack the performance specifically in low-resolution images and varying background conditions. We propose a coarse-to-fine pupil localisation method using a composite of machine learning and image processing algorithms. First, a pre-trained model is employed for the facial landmark identification to extract the desired eye frames within the input image. Then, we use multi-stage convolution to find the optimal horizontal and vertical coordinates of the pupil within the identified eye frames. For this purpose, we define an adaptive kernel to deal with the varying resolution and size of input images. Furthermore, a dynamic threshold is calculated recursively for reliable identification of the best-matched candidate. We evaluated our method using various statistical and standard metrics along with a standardised distance metric that we introduce for the first time in this study. The proposed method outperforms previous works in terms of accuracy and reliability when benchmarked on multiple standard datasets. The work has diverse artificial intelligence and industrial applications including human computer interfaces, emotion recognition, psychological profiling, healthcare, and automated deception detection.

## 1. Introduction

Detection and localisation of the objects within images or real-time video frames is considered an essential task in various computer vision algorithms [1]. Various studies have addressed the detection and tracking of facial landmarks including the iris and pupil which has various applications—particularly, eye gaze estimation for human–machine interfaces. The control of assistive devices for disability [2], driver safety improvements [3,4], the design of diagnostic tools for brain diseases [5], cognitive research [6], automated deception detection system (ADDS) [7], and academic performance analysis [8] are some examples of such applications. 

Research studies for the eye detection and eye tracking mostly focus on the iris and pupil localisation. Once the coordinates of pupils are determined, it can be used for the eye tracking, gaze estimation, and eye movements within the images and video frames [6]. Eye images can be characterised by the intensity distribution of the iris, pupil, and the cornea, in addition to their shapes. It should be noted that various aspects can influence the appearance of the eye including the viewing angle, ethnicity, head position, eye colour, light conditions as well as the texture, eye state (e.g., half closed, fully closed) and current well-being [6]. 

Overall, eye detection techniques can be classified as shape-based, feature-based, appearance-based, and hybrid methods. In the shape-based methods, open eyes are described by their shapes, including the pupil and iris contours as well as shape of the eyelids [9,10,11]. For the feature-based methods, the objective is to identify the local features within the eye that are less sensitive to the varying illumination as well as viewpoint [12,13,14,15]. Appearance-based methods depend upon detecting and tracking of the eyes using the photometric look, which is characterised by colour distribution and filter responses to eyes and their surroundings [16,17,18]. The hybrid methods aiming to combine various techniques to mitigate the particular disadvantages of these methods [19,20]. 

Standard methods in gaze estimation are based on corneal reflections that needs an accurate localisation of the pupil centre as well as the glints [21]. Pupil and glints localisation algorithms are usually based on image processing such as morphological operators for the detection of contour [22] and intensity threshold identification followed by the fitting using ray-based ellipse [23]. The topography-based hybrid method is introduced in [24], which uses series of filters for the iris centre estimation. However, these techniques assume that the pupil exists in the darkest area of the input image and may susceptible to varying illumination conditions that might require manual tweaking to the threshold parameters [25].

There are four main eye movement behaviours that are likely to show different details related to cognitive efforts when responding to tasks including blinks, pupillary responses, fixations, and saccades [31]. Blinking represents the involuntary deed of opening and closing the eyelids. Pupillary responses are the changes in pupil size restrained by the involuntary nervous system. Fixation represents the collection of gaze points that are relatively stable and near in spatial and temporal vicinity. Saccade represents the rapid and small eye movements when moving from one object to another [31]. These four eye-movement behaviours reveal the details about cognitive efforts and therefore can be used as suitable inputs for designing the machine learning (ML) systems as illustrated in Table 1, which shows various supervised ML algorithms to predict categorical responses from the eye movements. 

In addition to conventional methods, existing works also utilise the deep learning (DL) approaches for the pupil detection while using hierarchical image patterns to enhance and eliminate artefacts with Convolutional Neural Networks (CNNs). For instance, [21] proposed the use of fully connected CNNs for segmentation of the entire pupil area in which they trained the network on 3946 video oscillography images. These images were hand annotated and generated within a laboratory environment. The authors claim that the proposed network enables them to perform elliptical contour detection, pupil centre estimation, and blink detection. More explicitly, pupil centres are predicted with a median accuracy of one pixel and gaze estimation accuracy is within 0.5 degrees. However, varying image resolution might provide different accuracy measures. More specifically, [32] indicated the eye tracking as an important tool that can have a range of applications from scientific research to the commercial sector. The authors show that the use of tracking software based on commodity hardware including tablets and smartphones allows these advanced technologies to be available for everyone. The system is called iTracker, which uses a CNNs model indicating 2.53 cm and 1.71 cm prediction error without calibration on tablets and smartphones respectively, which is reduced to 2.12 cm and 1.34 cm using calibration.

The research presented in [23] proposed a pipeline of two CNNs cascaded for pupil detection. The authors claim that their method outperforms state-of-the-art techniques with a detection rate up to 25% while avoiding computational complexity. To benchmark their proposed technique, 79,000 hand-labelled images were used in which 41,000 were complementary to existing images from the literature. A similar work is presented in Naqvi et al. [33], which indicate that automobile accident deaths could be minimised using drivers’ gaze region to provide their points of attention. In this respect, the authors suggest the use of DL for gaze detection with the use of near-infrared camera sensors. They incorporate driver head and eye movement into their study. Gaze estimation accuracy was benchmarked using a loosely correct estimation rate and strictly correct estimation rate in which the study claims achieving good accuracy when benchmarked with the previous gaze classification techniques. 

Recent work that uses the CNNs-based DL model for the pupil estimation [34] indicates around 70% accurate estimation, while the error threshold is within 5 pixels. However, this accuracy is limited to be used in real time—specifically, the applications that consider micro-movements within the eyes such as ADDS [7]. A similar work that uses CNNs for the pupil detection [23] indicates varying detection rates (70–90%) with respect to the tolerance level as the pixel error and dataset they employed for testing. The study outcomes clearly indicate the trade-off between the error tolerance level and accuracy measure. Furthermore, the performance metric used in these studies is not standard (i.e., the error as number of pixels) and might produce varying accuracy with respect to image size and resolution. In contrast to CNNs, [35] utilises the wavelet transform to extract the distinguishing features, while SVM is used for the pupil classification. This work indicates 88.79% of accurate pupil estimation on a benchmarked dataset while utilising the standard validation metric. Despite the variety of existing methods for the pupil localisation, further improvements are required in terms of a precise estimation for the pupil location. For instance, the DL-based pupil localisation and gaze estimation in [21] uses pixel distance to validate the performance, which is not a standard representation of the error in the case of varying resolutions. Furthermore, the validation is performed on a dataset containing artificially rendered images, which in most cases do not reflect the real-time dynamics. Likewise, [36] presented gaze estimation that utilises the DL-based facial landmarks detection following the image segmentation to identify the pupil within the input images. However, the 81% accuracy produced by the algorithm on a benchmark dataset indicates the lack of preciseness in pupil localisation that might lead to the incorrect gaze estimation. Furthermore, this study along with [23,24] utilises a static threshold while considering the pupil as the darkest area within the image that may be susceptible to various illumination conditions [25] and low-resolution images. Likewise, the use of static size kernels for the template matching to find out the best-matched candidate (i.e., the pupil in this case) within the image might causes local maxima. For instance, a smaller-sized kernel may cause attention to noisy details (i.e., local maxima), whereas a larger size may lead to mismatches and an incorrect estimation of pupil location [37].

In the proposed work, we introduce an efficient algorithm for the pupil identification within low-resolution images (and video frames) using a composite of DL and image processing algorithms. To clarify the novelty of this paper, the contributions are outlined as follows: (1) utilising the pre-trained DL model to identify the facial landmarks and extraction of desired eye frames within the input images; (2) unidirectional cascades of two-dimensional (2D) convolution is used to determine the pupil coordinates within the eye frames of varying characteristics; (3) an adaptive kernel size is used to deal with the varying size of input images (i.e., eye frame) during the template matching; (4) we used a dynamic threshold to identify the best matched candidate more reliably; (5) for the first time, we introduce a relative error metric to measure the standardised distance (i.e., error) between the estimated and actual pupil centres; (6) we validated the proposed methodology over multiple publicly available and benchmark datasets containing high diversity in gaze positions, participants background, lighting illuminations, image background, and a comparatively smaller size of eye frames.

The remainder of this paper is organised as follows. Section 2 entails the proposed methodology and algorithms. Section 3 presents the detailed experimental design and newly introduced evaluation metric. The statistical results and technical discussions are presented in Section 4 followed by a conclusion and future works in Section 5.

## 2. Proposed Method

**Figure 1:**
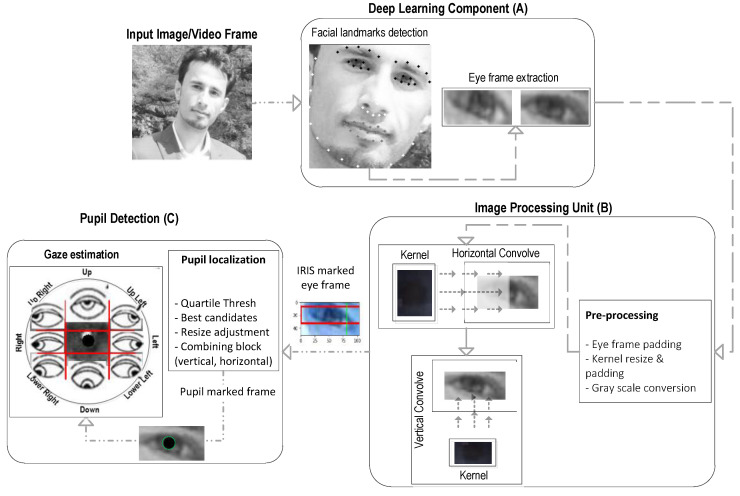
Sequential processing components of the proposed method comprising (**A**) deep learning (DL) library (i.e., Dlib-ml) for the eye-frame extraction, (**B**) computer vision algorithm for localising the potential iris and pupil candidates within eye-frames, (**C**) post-processing for the pupil coordinate measurement. In images, eye’s view is reversed (e.g., the left eye in an image is actually the right eye and vice versa).

The proposed pupil detection utilises a composite of techniques along with new algorithms while leveraging the DL-based facial landmark detection [38] to extract the eye information within an image/video frame. The existence of background noise and dark patches within the image frame and specifically prominent eyebrow parts are normally detected as pits that might cause mismatch for computer vision-based iris and pupil detection [24,37,39]. However, this issue can be resolved readily by utilising modern DL algorithms for a reliable face and eye-frame extraction from ordinary quality images or video frames. In the first step, we utilise the facial landmark detection to extract the desired segments containing only the eye frames (both left and right) from an input image. Then, we convolve the extracted eye frame with a pre-defined kernel in horizontal and vertical directions to identify the iris and pupil respectively within the eye frame. We adapt the kernel size dynamically with respect to the varying eye-frame size to resolve the possible occurrences of local maxima being a false representation of best matched patches. We further define a dynamic threshold for the identification of the best-matched patch within the current eye frame to reduce the impact of noisy matches. Figure 1 shows the sequential processing in our work to identify the pupil coordinates within an input image/video frame. The major components are (1) DL-based eye-frame extraction, (2) image processing-based iris localisation, and (3) pupil detection, which are detailed in the following sub-sections. 

### 2.1. Eye Frame Extraction

The DL component utilises a well-known toolkit (Dlib-ml) [38] that can reliably identify the facial landmarks while producing extensive fiducial points (68 in total) on the face, including eye corners and eye lids, as shown in Figure 1A. We first extract the face rectangle from an image using Dlib-ml that not only removes the unnecessary portion of the input frame but also helps to eliminate the major noisy components that might exist in the background region of the image frame. Within the face region, we then note the identified extreme points (left, right, top, bottom) for eye corners and eyelids, which are used to crop the exacted eye frames within the identified face rectangle. This is one of the major advantages of using Dlib-ml, which reliably eliminates the unnecessary portion of an image and extracts the exact region of interest (i.e., eye frames in this case) from the input frame. Only the input images (or video frames) with exactly one face rectangle and two eye frames are considered as ‘valid’. The output of this component in form of eye frames (left, right) is processed further to identify the iris and pupil within the image.

### 2.2. Iris Segmentation and Pupil Localisation

Following the eye-frame extraction, a convolution function is applied for the template matching between a custom kernel and eye frame to localise the best-matching segment within the eye frame. Firstly, we built a custom kernel representing 100 iris frames (cropped from eyes frames) randomly chosen from the datasets described in Section 3. The advantage of a custom kernel over an ordinary black colour kernel is a more generalised representation of an iris for a diverse population and morphology characteristics (e.g., geometry, patterns within the iris, colour, etc.). Another common factor that can affect the template matching performance is the size of the template (i.e., kernel). A smaller sized kernel may draw attention to noisy details (i.e., local maxima), whereas a larger size may lead to mismatches and incorrect estimation [37].

(1) $$y\left[i,j\right]=\overset{\infty }{\underset{m=-\infty }{\sum }}\overset{\infty }{\underset{n=-\infty }{\sum }}K[m,n]\cdot E[i-m,\;j-n]$$

**Figure 2:**
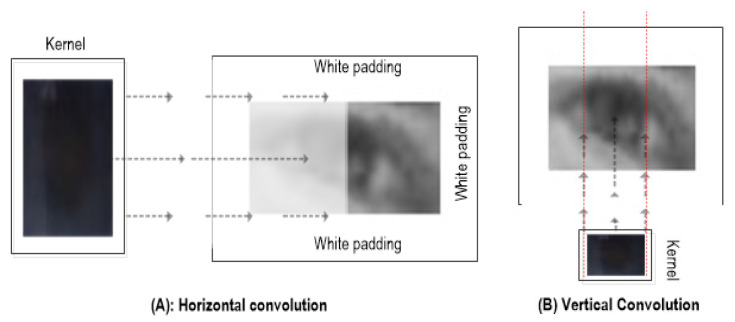
Horizontal convolution (**A**) and vertical convolution (**B**) between adaptive size kernel *K* and white outlined eye frame *E*.

To resolve this issue, the adaptive size kernel is employed using the interpolation and extrapolation techniques where the size (*w*_k_ × *h*_k_) varies with respect to the input frame size (i.e., eye frame). Furthermore, the eye frame (*E*) is padded with a rim of white pixels (see Figure 1 and Figure 2) to enlarge it enough so that the convolution kernel (*K*) fits inside the padded image to identify all the possible best matches (i.e., between kernel *K* and the overlapped eye-frame patches of a size similar to *K*)—more specifically, when the desired patch (i.e., iris) is located at extreme positions (e.g., looking in extreme left/right positions).

Equation (1) represents a 2D convolution function where *E* is the current eye frame (within the input image) to be convolved with the kernel matrix *K* resulting in *y* as the output image. The indices *i, j* and *m, n* represent the indices within the *E* and *K* matrices (i.e., image pixels), respectively.

In contrast to the ordinary technique of 2D convolution where kernel *K* slides along *E* with a fixed overlapping window (usually 1 pixel) in both horizontal and vertical directions, we perform comparatively simple and efficient convolutional steps (only one slide per horizontal and vertical directions), as shown in Figure 2. The reason behind an adaptive kernel selection is that the geometric features of the iris and pupil are considered approximately circular and black compared to the rest of the eye, with the pupil as the darkest segment. First, kernel height *h*_k_ is resized to eye-frame height (i.e., *h*_e_ = *h*_k_), and the width *w*_k_ is set to 0.4 of the eye-frame width. Then, the convolution function slides through *E* in the horizontal direction to determine the x-coordinate of the iris centre within the *E*. It compares the overlapped patches of *E* (*w*_k_ × *h*_k_) against *K* to calculate the matching scores at each horizontal stride (i.e., 1 pixel). The normalised correlation coefficient calculates a total matching score for the current patch in *E* using Equation (2).

(2) $$S\left(x,\;y\right)=\frac{\sum_{x\prime y\prime }(K^{\prime }\left(x^{\prime },y^{\prime }\right)\cdot \;\;E\prime \left(x+x^{\prime },y+y^{\prime }\right)}{\sqrt{\sum_{x\prime y\prime }K^{\prime }{\left(x^{\prime },y^{\prime }\right)}^{2}\cdot \sum_{x\prime y\prime }E^{\prime }{\left(x+x^{\prime },y+y^{\prime }\right)}^{2}}}$$

where S(x,y) is the matching score of the current overlap (*x, y*) between *K* and the *E* patch with a size equal to *K* (*w_k_* × *h_k_*). The summation in Equation (2) is performed over the *K* and *E* patch where *x′* = 0 …*w*_*k*−1_, *y′ =* 0 … *h*_*k*−1_. As the kernel height *h_k_* is aligned with height of the eye frame (i.e., *h_e_ = h_k_*), there are no vertical overlapping (i.e., no vertical overlapping/strides), which means that the kernel will only be able to move along *E* in the horizontal direction while computing the matching scores for overlapped patches in *E*. 

Once all the horizontal matching scores are calculated, the next step is to find the coordinates of the best matching segment. There have been several approaches to select the optimal match, but the candidate with maximum match has been commonly used in similar works [12,37,40]. However, it can easily cause local maxima, specifically in low-resolution images [37]. Likewise, using a pre-defined matching threshold can provide varying matching scores regarding the environment and can also mislead because of varying dynamics such as illuminations. We utilised quantile measure to select all the candidates (*M*) in the horizontal direction that cross the adaptive threshold of the 90th percentile of the matching scores sorted in ascending order; i.e., $M\;\in {SC}_{h}\;\mathrm{such}\;\mathrm{that}\;\forall \;SC_{h}>90\mathrm{th}\;\mathrm{percentile}\;\mathrm{of}\;\mathrm{sorted}\;SC_{h}$.

The mean of the horizontal (*x*-axis) coordinate of *M* selected patches is calculated using (3), which represents the x-coordinate of the top-left corner ($R_{x,y}$) of the final best-matched patch (i.e., estimated iris rectangle).

(3) Rx=∑i=1mMx (i)m

where *m* represents the total number of elements (i.e., best-matched candidates) in *M*, and $M_{x}$ is the horizontal coordinate of the corresponding best-matched candidates *M*. 

The iris rectangle *I* is identified using $R_{x}$ and kernel width *w_k_*, which is then used for the vertical convolution to identify the y-coordinate of iris centre. Similar to horizontal convolution-based matching, kernel height *w_h_* is resized to 0.4 of the height of *I* for overlapped stride matchings while keeping the width the same. Then, vertical convolution steps are performed to compute the matching score for *K* and the overlapped patches of *I* along the vertical direction only. The output matrices *SC_v_* contains all the corresponding matching scores for vertical convolutions between the *K* and *I* overlapped patches. The quantile measure is used in a similar way to select all candidates (*N*) in the vertical direction that cross the adaptive threshold of the 90th percentile of the matching scores sorted in ascending order, where $N\;\in {SC}_{v}\;\mathrm{such}\;\mathrm{that}\;\forall \;SC_{v}>90\mathrm{th}\;\mathrm{percentile}\;\mathrm{of}\;\mathrm{sorted}\;SC_{v}$. Then, the mean of the vertical (*y*-axis) coordinate of *N* selected patches is calculated using Equation (4), which represents the y-coordinate of the top-left corner ($R_{x,y}$) of the final best-matched patch (i.e., the estimated pupil rectangle).

(4) $$R_{y}=\frac{\sum_{i=1}^{n}N_{y}\;\left(i\right)}{n}$$

where *n* is the total number of elements (i.e., the best-matched candidates) in *N*, and $N_{y}$ is the vertical coordinate of the corresponding best-matched candidates *N*. 

Finally, the centre coordinates of the best-matched patches within *E* in the horizontal (*C_x_*) and vertical directions (*C_y_*) represent the pupil location along the *x*-axis and *y*-axis respectively and are calculated as:

(5) $$C_{x}=R_{x}+w_{k}/2,\;C_{y}=R_{y}+h_{k}/2$$

where *w_k_* and *h_k_* are the width and height of kernel *K*, respectively. Algorithm 1 summarises all the sequential steps involved in the proposed methodology to determine the pupil coordinate within an image frame.
**Algorithm 1:** Proposed algorithm for iris detection and pupil localisation in an image/video frame.**Inputs**: image/video frame *F*, a custom-defined kernel frame *K* **Output**: Pupil coordinates (*Cx, Cy*), iris rectangle (top-left; bottom-right) **STEP1**:-Initialise validation *Score = 0* for current *F*-Use *Dlib-ml* for the facial landmark detection within input frame *F*-Crop the face rectangle (*Face*) using the detected landmarks-IF count (*Face*) == 1 (i.e., exactly one face in an image is found)  - *Score ++*  - Extract the eye frames (*E_L_*, *E_R_*) for *left* and *right* eye  - IF count (*E_L_*, *E_R_*) == 2. i.e., exactly 2 eyes within the *Face* rectangle    ■ *Score ++*    ■ Goto STEP 2  - ELSE    ■ Mark it as invalid frame    ■ *Goto* STEP 1 for the next *F*-ELSE  - Mark it as invalid frame  - *Goto* STEP 1 for the next *F*
**STEP2:**
  - Foreach *eye frame E* in *E_L_*, *E_R_*    ■ Convert *E* into greyscale    ■ Outline *E* with white paddings    ■ Adapt the kernel *K height* to the *height* of *E* and *width* to 0.4*width(E)    ■ Convolve *K* with *E* by sliding *Horizontally* with a 1-pixel stride/sliding window    ■ Store the matching scores for overlapped *E* patches in a vector *SC_h_*    ■ Store the horizontal elements with high matching scores in lists *M* for $M\;\in {SC}_{h}\;\mathrm{such}\;\mathrm{that}\;\forall \;SC_{h}>90^{\mathrm{th}}\;\mathrm{percentile}\;\mathrm{of}\;\mathrm{sorted}\;SC_{h}$.    ■ Find the *top-left* of the best-identified iris rectangle by taking the mean (*µ*) of x-coordinates for *M* (i.e., $R_{x}$) using Equation (3)    ■ Find the iris rectangle *I*, using $R_{x}$ and *w_k_*    ■ *Goto* STEP3  - End Loop
**STEP3:**  - Adapt the kernel *K height* to 0.4*height(I) for vertical convolution  - Convolve *K* with *I* by sliding *Vertically* with a 1-pixel stride/window  - Store the matching scores for overlapped *I* patches in a vector *SC_v_*  - Find the elements with high matching scores (call them *N*) where $N\;\in {SC}_{v}\;\mathrm{such}\;\mathrm{that}\;\forall \;SC_{v}>90^{\mathrm{th}}\;\mathrm{percentile}\;\mathrm{of}\;\mathrm{sorted}\;SC_{v}$  - Find the *top-left* coordinate of the best-identified rectangle by taking the mean (*µ*) of y-coordinates of *N* (i.e., $R_{y}$) using Equation (4)  - Find the pupil centre C_x_, C_y_ by adding the width and height of *K* into $R_{x}$ and $R_{y}$ respectively using Equation (5).

## 3. Experimental Design

We conducted detailed experiments to validate the proposed methodology while using various datasets and validation metrics. We also performed a critical analysis based on various conditions and validated the proposed algorithm while considering the diversity in validation datasets as well as validation metrics. The following sections explain the validation datasets and metrics along with a detailed experimental design.

### 3.1. Datasets

To validate the proposed methodology and reliable performance measure, we used three different publicly available datasets. The first dataset is known as Talking-Face [41] and has been used in previous works [36]. This dataset contains 5000 video frames captured during an engaged conversation with a person for 200 s. The original objective of this dataset was to model the facial behaviour during a natural conversation. Data are captured with a static positioned camera with a frame size of 720 × 576 pixels. Every frame is annotated in a semi-automated manner containing 68 facial points, including the pupil coordinates. Following our validation check in Algorithm 1 (i.e., frames with exactly 2 eyes/frame) and removing the fully closed eyes (manually, we found 280 images) images, we are left with 4720 frames for the validation purpose. The dataset contains varying gaze positions, facial and body movements, diverse natural expressions, and variations in eye state (e.g., closed, open, half closed). However, because it is captured from an individual person, the diversity within the eye characteristics is very limited. In other words, there are no variations in terms of eye characteristics (e.g., iris or pupil colour, intensity, iris pattern, etc.) and hence, it is not very challenging for the algorithm validation.

In contrast to Talking-Face, we used the BIO-ID dataset [42], which is comparatively more challenging and has been used as a benchmark in various relevant studies such as [36,40]. The data were acquired from 23 different subjects during multiple sessions and have 1521 images in total containing varying gaze positions, illuminations, background scenes, eye features (e.g., eye colour, gender, ethnicity, iris size), camera focus, and hence eye-frame (and face rectangle) size. The interesting aspect of this dataset is the comparatively lower resolution (greyscale 384 × 288 pixels), which makes the validation of the pupil localisation algorithm more challenging but reliable. Besides, the dataset contains natural expressions such as images with half-closed eyes that further help to measure the validity of the proposed algorithm. Our algorithm detects only seven frames as invalid (i.e., not containing exactly two eyes), whereas we found 45 images (manually) with fully closed eyes that were excluded, resulting in 1469 images in the remaining dataset for validation purposes. 

Furthermore, we evaluated our method on a comparatively larger dataset known as GI4E [43] containing more diversity involving various morphology types (e.g., eye size, eye/iris features, gender, ethnicity, varying backgrounds and illuminations). It should be noted that despite higher resolution images (800 × 600 pixels), the size of the eye-frame rectangles is comparatively small. This is because of the larger distance of the capturing device from the subject, resulting in a lower ratio of the eye frame to the entire image. In other words, the whole frame covers more background pixels as compared to the actual face within the image, which makes the eye frame and hence iris/pupil localisation more challenging. The dataset is much more diverse, containing 103 subjects (each with 12 images) with 1236 total images involving 12 different gaze position. In addition, there are no open eyes or invalid frames in this dataset.

### 3.2. Validation Metrics

One of the important factors in validation of the pupil detection and proposed work is the metric we chose for the performance measure. This is because of the nature of the pupil localisation problem. For instance, the absolute error in the estimated pupil/eye centre and actual eye centre might vary with respect to image size/resolution. Hence, a standard distance measure such as Euclidean distance (ED) and/or R^2^ coefficient will not give a true representation of the accuracy measure. The authors in [42] introduced a relative error measure ($d_{eye}$) to deal with this issue, which has been utilised in various related works [36,37,42,44]. It uses the maximum of the estimated pupil coordinates distances from left and right eyes ($d_{l}$) and $(d_{r}$) respectively, between the actual eye centres ($C_{l}$, $C_{r}$) and the estimated ones (${\tilde{C}}_{l\;}$,${\tilde{C}}_{r}$) using Equation (6).

(6) $$d_{eye}=\;wec=\frac{max\left(\;∥{\tilde{C}}_{l}-C_{l}∥,\;\;\;∥\;{\tilde{C}}_{r}-C_{r}∥\;\right)\;}{∥C_{l}-C_{r}∥}$$

For the normalisation, the calculated distance is divided by the distance between two actual eye centres $∥C_{l}-C_{r}∥$, as shown in Equation (6). The normalisation factor makes the error measure independent of the image scale and hence eye-frame size. Furthermore, [36] used the best eye centre (bec) which utilises the minimum of the error between the estimated and actual centres as:

(7) $$d_{eye}=\;bec=\frac{min\left(\;∥{\tilde{C}}_{l}-C_{l}∥,\;\;\;\;∥{\tilde{C}}_{r}-C_{r}∥\;\right)\;}{∥C_{l}-C_{r}∥}$$

Although the *wec* (i.e., the worst eye centre) metric provides a relative error estimate, it is based on some assumptions such as ‘on average population, the distance between the inner eye corners is equal to width of a single eye of the corresponding subject’. Likewise, a relative error of $d_{eye}$ = 0.25 is considered as half of an eye width, which may not be valid in every case. Interested readers can get further details in [42]. 

To further deal with the metric generalisation issue, for the first time, we introduce a standardised error measure (*S_ED_*) as a function of distance between the estimated and actual coordinates within an eye frame. It calculates the relative distance as a percentage of the total possible ED (i.e., error) between the actual and estimated pupil coordinates. The *S_ED_* measure interprets the error within the single eye frame without depending on the second eye or interpupillary distance used in other related works. Besides, the *S_ED_* metric can measure the relative error regardless of image/face or eye-frame size and hence the image resolution. Mathematically, the proposed *S_ED_* is defined as:

(8) $$s_{ED}=\frac{\sqrt{{\left(Cx_{e}-Cx_{a}\right)}^{2}+{\left(Cy_{e}-Cy_{a}\right)}^{2}}}{\sqrt{{\left(x_{min}-x_{max}\right)}^{2}+{\left(y_{min}-y_{max}\right)}^{2}}}\times 100$$

where $Cx_{e},Cx_{a}$ represents the estimated and actual pupil horizontal coordinates, respectively, and $Cy_{e},Cy_{a}$ represents the estimated and actual pupil vertical coordinates, respectively. The $x_{min},y_{min}$ are the coordinates of the nearest corner of the eye frame (usually the top left corner), whereas $x_{max},y_{max}$ are the coordinates of the farthest corner of the eye frame (usually the bottom right). The numerator in Equation (8) represents the error (in terms of pixels) between the actual and estimated positions, whereas the denominator is the total possible error and is used as a normalisation factor. The resulting *S_ED_* gives the percentage error representing a standardised distance between the actual and estimated pupil positions in pixels, which is not affected by the image size and resolution. In addition, to evaluate the pupil detection techniques, the proposed standardised distance measure can also be useful for other related works such as object localisation, image segmentation, and object tracking etc. 

In summary, a comprehensive comparative analysis is performed to evaluate the proposed methodology using the aforementioned metrics including *wec*, *bec*, and *S_ED_* along with other standard accuracy measures including the ED, absolute mean difference, and R^2^ (coefficient of determination).

## 4. Results and Discussions

Following the experimental design, performance of the proposed pupil detection approach is evaluated using various gold standards, validation metrics, and benchmarked datasets. As discussed in the experimental design, it is important to use appropriate evaluation methods due to the nature of the problem. To maintain the reliability in our performance measure, we utilised different metrics as well as the newly introduced *S_ED_* in this work. 

Table 2 summarises the results achieved from the proposed approach using *wec* and *bec* metrics, which have been used in recent similar works [35,36,37,42,43,44]. We are specifically interested in the *wec* measure when error ≤0.05, which indicates the model estimation within the pupil diameter (i.e., more restricted). The best accuracy achieved by the proposed method is 97.1% while tested over the Talking-Face dataset, which outperforms the 89.59% presented in a recent work [36] that uses the same dataset. The high accuracy is expected because of the comparatively less challenging nature of the dataset (see Section 3.1). Firstly, the dataset contains high-resolution images. Secondly, the data are captured from only one person; hence, generalised iris and eye patterns are easily detected. It is important to note that despite the dataset being collected from single person, it contains high variations in terms of gaze, head movements, facial expressions, and sufficient quantity (i.e., 5000 images) with annotated pupil coordinates. On the other hand, the proposed method achieves 100% *wec* accuracy while testing for an error threshold of ≤0.1, indicating the robustness of the proposed methodology. This means that the model estimation regarding pupil coordinates is within the iris in all cases (i.e., 5000 images). Overall, the proposed method outperforms the most recent works in relatation to pupil localisation [36] while evaluating the Talking-Face dataset.

To further evaluate the model performance, the BIO-ID dataset is used, which contains various subjects as well as high variations in gaze, head pose, and body movements. Furthermore, the image quality (i.e., resolution) is comparatively lower (i.e., 286 × 384), which makes it more challenging when focusing the identified eye frame and/or iris/pupil within the image. In addition, a large proportion of the entire image contains background rather than the face itself, which makes the dataset more challenging, as addressed by the previous works [37]. Despite the associated challenges, the proposed approach shows robust pupil estimations, as shown in Table 2. The model indicated significant improvements with a 94.5% *wec* measure with an error threshold ≤0.05 when benchmarked with the works of [36] and [39] of 81% and 84%, respectively. Furthermore, the model indicated 100% accuracy when evaluated for an error threshold ≤0.1, which means that pupil localisation is within the iris in all cases (i.e., 1521 cases in total). Despite the 100% *wec* and *bec* accuracy for an error threshold ≤0.1, the main focus is to maximise the *wec* accuracy (which is the most challenging) with a minimum error threshold (i.e., ≤0.05) to restrict the model estimation within the pupil diameter.

**Figure 3:**
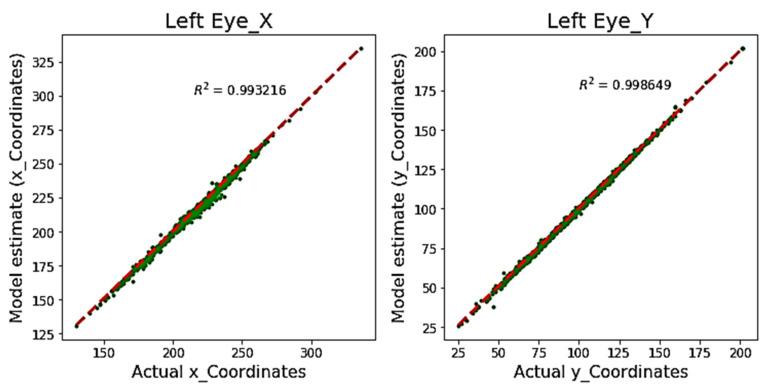
Comparison of estimated pupil coordinates (pixel position) using the proposed model, with actual annotated coordinates (BIO-ID dataset) using an R-squared error.

Figure 3 shows the R^2^ coefficient for the proposed model tested on the BIO-ID dataset. It can be observed that the *x*-axis and *y*-axis estimated coordinates are almost overlapping the actual annotations with R^2^ values of 0.993 and 0.998 for the *x*-axis and *y*-axis, respectively. Although R^2^ is a well-known statistical measure to determine the goodness-of-model fit, it might not be effective for validating the model estimation in pupil detection or similar problems because of the varying error rate with respect to the image size (and resolution).

Table 3 summarises the comparative results from various previous works while weighted over the challenging BIO-ID dataset using the *wec* metric with varying thresholds. It can be noticed that the proposed model outperforms (94.5%) all the previous works specifically with the most restricted error threshold: ≤0.05. Recent works that uses a similar approach [36] achieved accuracies of 80.9% and 82.5% [37] with e ≤0.05, whereas the best accuracy of 88.79% was indicated by [35], which is significantly lower than the proposed method. The research study in [21] presented a robust technique for the pupil localisation and gaze estimation; however, the measured performance is not standard (i.e., it uses the mode of pixel distance, which is not the true representation of error with varying resolutions). Furthermore, the validation is performed on a different dataset containing artificially rendered images, which in most cases do not reflect the real-time dynamics.

Besides the Talking-Face and BIO-ID datasets, we evaluated the performance of our proposed approach on another challenging dataset: GI4E. It can be noted from Table 2 that our model produces 95.05% *wec* and 98.71% *bec* accuracy respectively with a critical threshold of *≤*0.05. While most of the existing works used BIO-ID as a benchmark dataset, some of them also used GI4E to evaluate their techniques. For instance, a recent study on eye centre localisation [24] reported 93.9% *wec* accuracy on the GI4E dataset, which is slightly lower than our approach (i.e., 95.05%). However, their accuracy decreased to 881.2% when tested on the BIO-ID dataset. This indicates the robustness of our proposed approach for pupil detection in varying datasets containing diversity regarding eye colour, gaze position, facial emotions, and real movements. Similarly, [45] indicated 89.28% *wec* accuracy on the GI4E dataset, which is significantly lower than the proposed approach. A clustering-based approach [46] produced a mean pixel error of 2.73 pixels as compared to our proposed model, which produced 1.7 pixels when validated on the GI4E dataset. However, it is important to note that this metric does not represent a standard accuracy measure, as described in Section 3.2.

In addition to *wec,* [24,40] used an average point-to-point error (m_e17_) with the interocular distance between the left and right eye pupil. A recent work [21] that uses DL to localise the pupil and estimate the gaze position also employed the median of absolute difference in the *x*-axis and *y*-axis. However, variations in image size, zoom-in/out due to body/head movements, and/or camera positions might affect the mean difference in corresponding error estimate, resulting in variations in accuracy measure. The *wec* metric, which has been used extensively in related works such as [24,36,37,38,39,44,45], gives a comparatively better indication of the performance measure. However, these metrics measure the performance in terms of coordinate estimation within the pupil/iris diameter with a varying error threshold, as shown in Table 2. In addition, it is based on relative error assumption ($d_{eye}$ = 0.25) as half an eye width, which may not be true in every case. Therefore, model estimations and performance measurements (specifically in the pupil localisation task) need to be evaluated using a more standard metric representing the distance between estimated and actual pupil coordinates.

To overcome this issue, we first time introduce a standardised Euclidean distance (*S_ED_*), which represents the percentage distance error as ED using Equation (7) (see Section 3.2). The error represents the displacement between the actual and estimated pupil coordinates as a percentage of the whole image size (i.e., eye frame) in terms of the number of pixels. The major advantage of *S_ED_* is a standard representation of the error that can be used to measure the accuracy regardless of image size and resolution, which is not the case in *wec*, m_e17_, and other metrics used in most of the existing studies. Table 4 presents the comparative analysis of proposed model estimations in terms of mean pixel difference in each axis for both eyes (left and right), the R^2^ coefficient, the ED between the centre of the estimated and actual pupil coordinates, and the newly introduced *S_ED_*. The proposed method indicates 1.04 and 0.57 absolute pixel errors on the *x*-axis and *y*-axis, respectively (i.e., 0.8 on average for both) as compared to 2.91 in [46] on the BIO-ID dataset. Similarly, a DL-based model in [23] indicated their optimal performance with a pixel error >10. However, they used different datasets, which in the case of high resolution is not comparable with the proposed method and clearly indicates the need for a standard metric similar to *S_ED_*.

It can be analysed that the model performs comparatively better for the Talking-Face and BIO-ID datasets as compared to the GI4E dataset based on the corresponding properties (as discussed in Section 3). However, there are several crucial aspects to be noted in each case. First, in contrast to the *wec* measures in Table 2, the ED (c_a,_ c_e_) error in Table 4 for Talking-Face is 1.96, which is higher than the other two datasets (1.43 and 1.70 for BIO-ID and GI4E, respectively), despite the high quality and fewer variations in the former case. This is because the size of the images in the Talking-Face dataset is comparatively larger than those in the other datasets, and consequently, the ED (c_a,_ c_e_) error as well as absolute error (µ|x_a_−x_e_|, µ|y_a_−y_e_|) in each axis is also high. However, the results from these metrics (i.e., ED, µ|x_a_−x_e_|, µ|y_a_−y_e_|) do not align with the results in Table 2 (*wec* measure) and therefore do not reflect the true measure of the standardised difference between estimated and actual pupil coordinates. In contrast, *S_ED_* provides a more generic and standard representation of error between the actual and estimated coordinates as a percentage of the eye rectangle size. The *S_ED_* error for the Talking-Face dataset is 2.49%, which is less than the 3.98% and 3.87% errors of the BIO-ID and GI4E datasets, respectively, and it also aligns with the *wec* outcomes in Table 2. As mentioned earlier, *S_ED_* represents a standardised distance (i.e., pixels) using the current eye frame without depending upon the second eye or interpupillary distance, which is not the case in *wec* measurement. Furthermore, *S_ED_* interprets the error in terms of pixel distance without using any thresholds (as in the case of *wec*) and can be utilised as a standard metric to evaluate the true performance of such models in similar problems.

**Figure 4:**
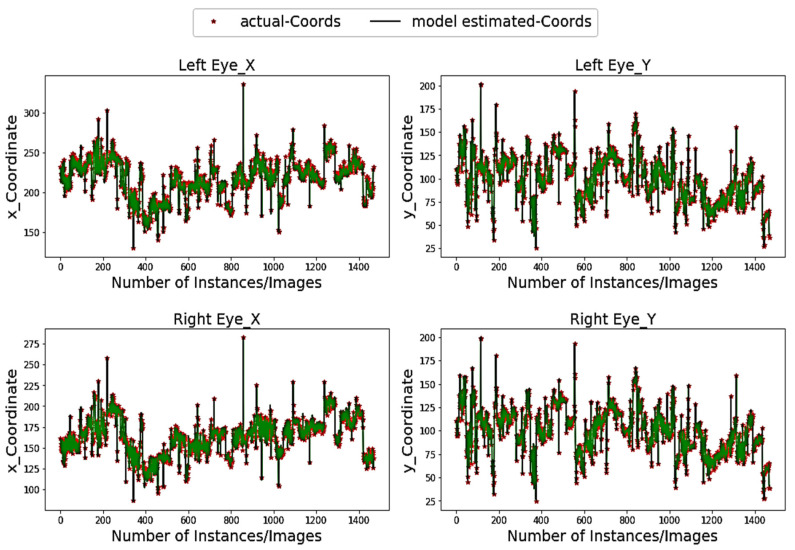
Pupil coordinates estimations (green color) vs. actual (red) coordinates within the BIO-ID dataset.

Figure 4 demonstrates the pupil estimation performance of the proposed model for both the left and right eye (*x*-axis and *y*-axis) on the BIO-ID dataset. The model indicates a perfect overlapping for both axes and more specifically at the peak positions, which represent the extreme pupil and/or iris positions looking extremely left or right as well as the top or bottom positions. One of the reasons of such robust overlapping is the use of white paddings in our model, which helps the adaptive kernel achieve maximum overlaps at extreme positions resulting in appropriate matching candidates during horizontal and vertical cascades.

**Figure 5:**
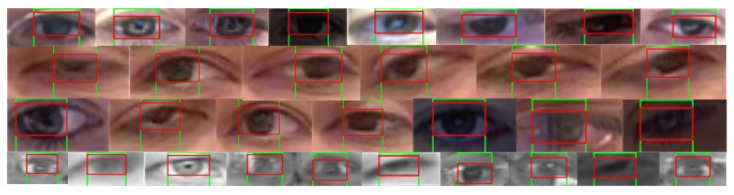
Horizontal and vertical convolution-based pupil coordinates localisation (in randomly selected images from the BIO-ID, GI4E, and Talking-Face datasets) for dynamic conditions such as gaze position, eye colour, intensity, noise interference, eye size, and image resolution.

As discussed earlier, a custom kernel might help for the optimal representation of iris diversity. Additionally, the adaptation of kernel size regarding the eye frame and dynamic threshold for best candidate selection further improves the reliability of our method specifically in dynamic conditions. Figure 5 demonstrates various test cases of iris/pupil detection using the proposed methodology for diverse eye properties and varying environmental conditions (e.g., patterns, gaze direction, varying background, half/full closed eyes, colour, intensity, illuminations, resolution, pupil/iris size, gender, ethnicity, etc.). It indicates the robustness of model estimations in both horizontal and vertical convolutions specifically at extreme positions (such as left/right corners, top right, half-closed, etc.)

Primarily, the proposed method is leveraging the pre-trained Dlib-ml that can locate the facial landmarks efficiently and reliably. It helps to filter out the unnecessary background segments within the input image as well as irrelevant facial components, excluding the desired regions that contain exact eye frames. Secondly, the proposed method uses efficient algorithms to adapt the kernel size in accordance with the eye frame and pad the eye frame with white surrounding pixels, which further reduces the probability of selecting noisy matched candidates, as mentioned by [36,37]. The use of a quantile-based dynamic threshold to identify the best-matching patch further enhances the reliability of the proposed algorithm (e.g., the outcomes in Figure 4 and Figure 5).

**Figure 6:**
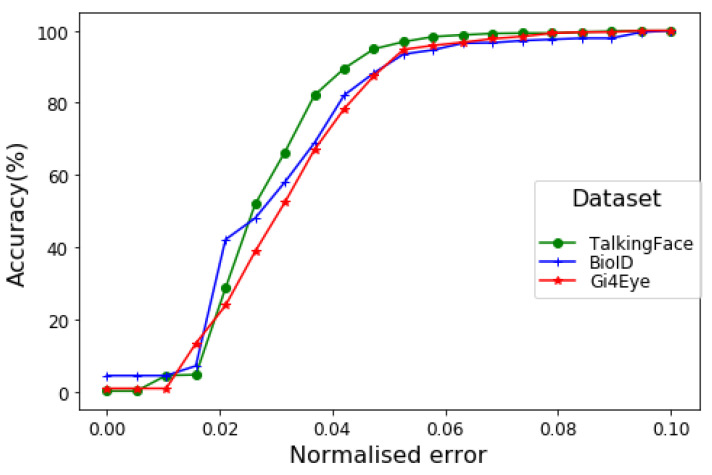
The *wec* measure for different datasets using the proposed method.

Figure 6 shows the performance of the proposed method for pupil coordinate estimation using the BIO-ID dataset while varying the error threshold to measure the mean *wec* for both eyes. The visualisation indicates accuracy over 90% in all cases (i.e., dataset) while considering the strict constraint of e ≤0.05. More explicitly, the model indicates that in over 97% of cases with high-resolution images/videos (which are ordinary for current technological advancement), the error in estimated pupil position is less than the diameter of the pupil itself. Even in the worst-case scenario (i.e., small-size eye frames in the GI4E dataset), the model achieves above 95% accuracy. 

**Figure 7:**
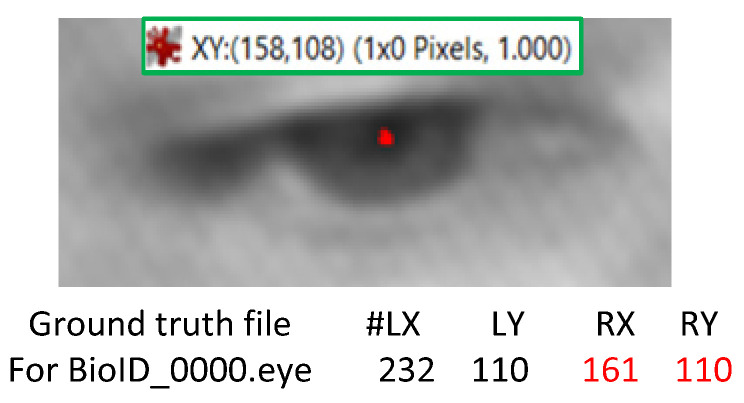
Example of annotation error in the BIO-ID dataset.

It is also imperative to mention that some annotation errors may slightly influence the performance measure, even though this is observed in very few cases. For instance, Figure 7 indicates the eye centre coordinates annotations in the BIO-ID dataset (R_x_:161, R_y_:110) provided by [42] for the right eye of subject BioID_0000.eye. However, the correct values are R_x_:158 and R_y_:108 (refer to Figure 7), which indicate approximately a 2-pixel difference in each axis. This is significant for micro-movements estimation and would affect the model performance substantially (e.g., *wec*, *S_ED_*).

Finally, it can be noted that the proposed model performs initial checks on the current frame quality to assure the existence of exactly two eyes (Algorithm 1) within the identified face rectangle. However, additional constraints can further improve the accuracy, specifically, in real-time scenarios and video stream data. For instance, [36] used the DL model to identify blinking eyes, which can further improve the accuracy of the proposed model while filtering out the images/frames without distinctive iris/pupils (i.e., separating the closed eyes not to be analysed for pupil localisation). Additional post-processing constraints such as symmetry constraints over the estimated pupils’ coordinates in both eyes might improve the gaze estimation accuracy. This might be useful to improve the eye-state information extraction approaches such as that used in [7] for the deception detection through facial micro-gestures.

## 5. Conclusions and Future Works

We proposed a novel pupil estimation method utilising the deep learning-based facial landmark detection and an image processing algorithm to determine the eye centre within an image frame. Reliable extraction of the eye frames within the input image is one of the major advantages of using Dlib-ml. This eliminates most of the background and irrelevant segments of the image, which helps to identify the target segment using intelligent image processing. We developed a customised iris kernel using multiple images from various datasets for its generalised representation. Then, the iris kernel is convolved with eye frame in two stages (horizontal and vertical) such that no nested strides are performed by the convolution function. The white paddings surrounding the kernel as well as the eye frame proved very helpful for template matching between the kernel and overlapped eye patches, specifically for the extreme eye positions (e.g., left/right corners). In addition, utilising a dynamic threshold for identifying the best-matched patch further contributed to the reliability of our method.

Compared to several state-of-the-art pupil detection methods, the proposed approach indicated significant improvements in pupil estimation accuracy, specifically with lower-resolution images and minimum error thresholds. We also introduced a standardised distance metric to measure the relative error in model estimation. This metric can be used regardless of image size and resolution, which is not the case with most of the existing validation metrics used in similar works. In future works, the proposed method will be utilised along with eye-blink detection models to determine eye gaze, in particular for infraduction iris positions. Our method can be useful in various computer vision applications, specifically the one requiring precise pupil and eye centre estimation. For instance, the eye-related feature extraction in [7] can be replaced with our method to extract the more reliable and micro-level movements within the eyes to distinguish truthful from deceptive behaviour. More explicitly, this work is expected to direct several application areas such as human–computer interfaces, gaze estimation, emotion recognition, psychological profiling, fatigue detection, healthcare, visual aid, and automated deception detection. 

## Figures and Tables

**Table 1:** Eye movements and classification algorithms.

Reference	Model	Aims and Feature Used
[26]	Hidden Markov model	Use of fixation count, fixation durations to distinguish between expert and novice participants
[27]	Multi-layer perceptron (MLP)	Use pupil size and point-of-gaze for predicting the users’ behaviours (e.g., word searching, question answering, looking for the most interesting title in a list)
[28]	Naïve Bayes classifier	Use of fixation duration, mean, and standard deviation to identify various visual activities (e.g., reading, scene search)
[29]	MLP	Use of pupil dilation, gaze dispersion to classify various tasks on decision making
[30]	Decision tree, MLP, support vector machines (SVM), linear regression	Use of fixation rate, fixation duration, fixations per trial, saccade amplitude, and relative saccade angles to identify eye movements to predict visualisation tasks

**Table 2:** Performance analysis of the proposed model using *wec*, *bec* with varying error thresholds.

Dataset	*Wec* (%)		*Bec* (%)	
	Error ≤ 0.05	Error ≤ 0.1	Error ≤ 0.05	Error ≤ 0.1
BIO-ID	94.5	100	98.34	100
Talking-Face	97.10	100	99.7	100
GI4E	95.05	100	98.71	100

**Table 3:** Performance comparison between previous works based on the *wec* measure using the BIO-ID dataset.

*wec* % Accuracy with Varying Error (e) Threshold				
Methods	e ≤ 0.05	e ≤ 0.1	e ≤ 0.15	e ≤ 0.2
[24]	81.1	94.2	96.5	98.5
[35]	88.7	95.2	96.9	97.8
[36]	80.9	91.4	93.5	96.1
[37]	82.5	93.4	95.2	96.4
[39]	84.1	90.9	93.8	97.0
[40]	57.2	96.0	98.1	98.2
[42]	38.0	78.8	84.7	87.2
[44]	47.0	86.0	89.0	93.0
[45]	85.8	94.3	96.6	98.1
Proposed Model	94.5	100	100	100

**Table 4:** Comparing model estimations using newly introduced *S_ED_*, Euclidean distance (ED), R^2^, and absolute error metrics.

Dataset	µ|x_a_−x_e_|	µ|y_a_−y_e_|	R^2^_x	R^2^_y	ED(c_a,_ c_e_)	%ED(c_a,_ c_e_)
BIO-ID	1.04	0.57	0.993	0.998	1.43	3.98
Talking-Face	1.23	0.97	0.990	0.956	1.96	2.49
GI4E	1.32	0.71	0.996	0.999	1.70	3.87
